# Multi-Omics Analysis of the Immune Effect of the Engineered Exosome Drug Delivery System in Inducing Macrophage Apoptosis

**DOI:** 10.3390/pharmaceutics17040494

**Published:** 2025-04-08

**Authors:** Wei Xiang, Zhoujun Zhu, Qisong Shang, Parhat Yasin, Yuanyuan Wu, Xinghua Song

**Affiliations:** 1 Department of Spine Surgery, The Sixth Affiliated Hospital of Xinjiang Medical University, Urumqi 830011, China; xiang_witsparkleia@163.com (W.X.); drshang3513@163.com (Q.S.); parhatpiko@zju.edu.cn (P.Y.); wyy1042962884@163.com (Y.W.); 2 Department of Joint Surgery, The Sixth Affiliated Hospital of Xinjiang Medical University, Urumqi 830011, China; drzzj1004@163.com

**Keywords:** engineered exosomes, rifapentine, macrophage, transcriptome sequencing, proteomics

## Abstract

**Background:** In this study, exosomes were engineered with anti-CD47 antibody and loaded with rifapentine to improve their ability to target macrophages for drug delivery. **Methods:** Exosomes from RAW264.7 cell supernatant were extracted by differential centrifugation, antibody-modified, and drug-loaded ultrasonically. After co-culturing with macrophages, transcriptomics and proteomics screened differentially expressed genes and proteins. Western Blot identified macrophage polarization, ELISA detected inflammatory indicators, and an apoptosis kit was used for fluorescence staining. **Results:** Transcriptome sequencing showed that 406 genes in the macrophages changed significantly, with pathways like TNF and NF-κB. Proteomics identified 7478 proteins, 433 with significant differences. Western Blot indicated M1 polarization. Fluorescence staining showed apoptosis in the antiMExo-RIF group. **Conclusions:** The study provides multi-omics evidence of the immune mechanism of the engineered exosome drug delivery system in inducing macrophage apoptosis, revealing potential molecular mechanisms and the great potential use of engineered exosomes in treating macrophage-related diseases.

## 1. Introduction

Tuberculosis, an ancient and intractable infectious disease caused by Mycobacterium tuberculosis, continues to pose a significant public health threat globally [1]. According to the latest data from the World Health Organization, there are still a large number of new cases every year, and the emergence of drug-resistant strains has made the treatment situation even more severe [2]. Macrophages, as a crucial line of defense in the body’s innate immunity [3], play a central role in the infection process of Mycobacterium tuberculosis [4]. During the complex interaction between Mycobacterium tuberculosis and macrophages, macrophages exhibit different polarization states, which have a decisive impact on the development of the disease [5]. M1 macrophages, usually polarized under the stimulation of interferon-γ (IFN-γ) and lipopolysaccharide (LPS), can secrete a variety of pro-inflammatory cytokines such as inducible nitric oxide synthase (iNOS), interleukin (IL-1), and tumor necrosis factor-α (TNF-α), and resist pathogen invasion by releasing antibacterial substances such as reactive oxygen species (ROS) and reactive nitrogen intermediates (RNI) [6]. However, Mycobacterium tuberculosis has evolved a variety of escape mechanisms that can inhibit the M1 polarization of macrophages, thus surviving and multiplying safely within macrophages [7].

Exosomes, as extracellular vesicles with a diameter of approximately 30–150 nm, are secreted by a variety of cells into the extracellular environment. They encapsulate bioactive substances such as proteins, nucleic acids, and lipids inside and possess the ability to transmit information among cells. In recent years, exosomes have emerged in the field of drug delivery due to their unique advantages such as their natural nanoscale size, good biocompatibility, low immunogenicity, and the ability to cross biological barriers [8]. Through engineering modifications of exosomes, for example, by modifying specific targeting molecules on their surfaces or loading therapeutic drugs, the targeting ability towards specific cells and the therapeutic effect can be significantly improved.

In recent years, through research on cell surface molecules, many targets that can be used to regulate the targeting ability of exosomes have been discovered. Among them, CD47, as a transmembrane glycoprotein widely expressed on the surfaces of various cells, plays an important role in immune recognition and regulation [9,10]. Under normal physiological conditions, CD47 interacts with the signal regulatory protein α (SIRPα) on the surface of macrophages, transmitting a “don’t eat me” signal, thereby inhibiting the phagocytosis of self-cells or foreign substances by macrophages [11]. This mechanism provides a theoretical basis for the modification of exosomes to target macrophages. By using the anti-CD47 antibody to engineer exosomes, it is possible to change the interaction mode between exosomes and macrophages, enhance the targeting ability of exosomes to macrophages, and then achieve more efficient drug delivery [12]. Moreover, exosomes have the characteristics of “homing and tropism” to their tissues or cells of origin, so the use of macrophage-derived exosomes in this study for the construction of drug-carrying systems can indirectly further enhance their macrophage-targeting properties.

Based on the above background, this study has innovatively constructed an engineered exosome drug delivery system, aiming to take advantage of the excellent properties of exosomes to precisely deliver anti-tuberculosis drugs into macrophages. It is expected that by inducing the polarization of macrophages towards the M1 direction and activating relevant inflammatory pathways, macrophage apoptosis can be ultimately induced, thus breaking the survival shelter of Mycobacterium tuberculosis within macrophages and opening up new avenues for the development of novel and efficient anti-tuberculosis treatment strategies. Through a series of multi-omics analyses and cell function experiments, this study focused on exploring the regulatory mechanism of the engineered exosome drug delivery system on the immune effect of macrophages, providing a brand new theoretical basis and practical guidance for the treatment of tuberculosis.

## 2. Materials and Methods

### 2.1. Extraction, Modification, Drug Loading and Characterization of Exosomes (Scheme 1)

#### 2.1.1. Exosome Extraction

The culture conditions for RAW264.7 cells are as follows: Dulbecco’s modified Eagle medium (DMEM) high-glucose (4.5 g/L glucose), supplemented with 10% fetal bovine serum (FBS) and 1% penicillin–streptomycin, maintained in a humidified incubator at 37 °C with 5% CO_2_ under saturated humidity. Before collecting the culture supernatant of macrophages, the cells should be cultured in exosome-free medium to eliminate the influence of serum-derived exosomes.

**Scheme 1:**
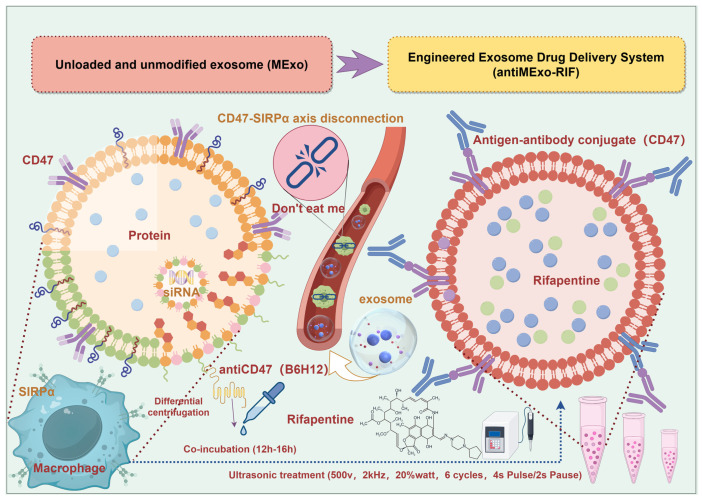
Schematic diagram of the preparation steps of the engineered exosome drug delivery system and the targeting principle of macrophages (By Figdraw, ID:ITSWS9f8e8).

The collected cell culture supernatant is subjected to low-speed centrifugation, typically at 300× *g* for 10 min, to remove cells and larger cellular debris. This is followed by medium-speed centrifugation at 2000× *g* for 10–30 min to eliminate smaller cellular debris and large vesicles. Subsequently, ultracentrifugation is performed at 10,000× *g* for 30 min to remove medium-sized membrane vesicles and protein aggregates. Finally, ultracentrifugation is carried out at 100,000× *g* at 4 °C for 70 min to pellet exosomes. The supernatant was carefully discarded, and the precipitate was resuspended with an appropriate amount of phosphate buffers (PBS). Subsequently, ultracentrifugation was carried out again at 4 °C with the same rotation speed and time to further purify the exosomes. Finally, the obtained exosome precipitate was resuspended with a small amount of PBS and stored at −80 °C for later use.

#### 2.1.2. Exosome Engineering Modification

The purified exosomes were incubated with the diluted (1:1000) anti-CD47 antibody (B6H12) at 4 °C for 12–16 h to ensure that the antibody had sufficient time to bind to the exosomes. Meanwhile, a control group was set up, in which the exosomes were only incubated in the same reaction buffer (Phosphate buffers, PBS) without the addition of the antibody.

#### 2.1.3. Exosome Drug Loading

First, rifapentine was dissolved (dimethyl sulfoxide, DMSO) and mixed with exosomes at a ratio of 1:1. After ultrasonic treatment (500 v, 2 kHz, 20% watt, 6 cycles, 4s Pulse/2s Pause) using the ultrasonic method, the mixture was allowed to stand for one hour. Subsequently, the unloaded drugs were separated by centrifugation through an ultrafiltration tube (100 KD). The precipitate was collected and resuspended to obtain the exosome solution loaded with rifapentine.

Macrophage exosomes (MExo) and the engineered exosome delivery system (antiMExo-RIF) were prepared by the above methods.

#### 2.1.4. Characterization of the Engineered Exosome Drug Loading System

Transmission electron microscopy (TEM) (HITACHI, HT7800/HT7700, Tokyo, Japan): An appropriate amount of the exosome suspension was dropped onto a copper grid and negatively stained with a negative staining solution. Then, the morphology of the exosomes was observed under a transmission electron microscope. Typical exosomes are cup-shaped or round with a double-membrane structure.

Nanoparticle tracking analysis (NTA) (Particle Metrix, ZetaView, Hallbergmoos Germany). Using a nanoparticle tracking analyzer, the exosome suspension was diluted to an appropriate concentration, and then its particle size distribution and concentration were analyzed. According to the NTA results, it was confirmed that the particle size of exosomes was mainly distributed between 30 and 150 nm, and the concentration information of exosomes was obtained at the same time.

Western blot (WB). The concentration of exosomes and protein extracted from cell samples was determined using the BCA assay. Protein samples were loaded into the wells of polyacrylamide gels (PAGE gels). Under an electric field, proteins migrated through the gel based on molecular weight for separation. The separated proteins were then transferred from the gel onto PVDF or nitrocellulose membranes, typically via electrophoretic or semi-dry transfer. The membranes were blocked with blocking buffer (5% skimmed milk) to reduce non-specific antibody binding. Subsequently, the membranes were incubated with primary antibodies specific to target proteins for 12 h, followed by washing to remove unbound primary antibodies. Horseradish peroxidase (HRP)-conjugated secondary antibodies were then applied for 2 h, and signals were detected using a chemiluminescence imager. The details of antibodies used are as follows: GAPDH (SanYing, #60004-1-Ig, 1:10,000, Hangzhou, China), CD63 (Abcam, #Ab216130, 1:1000, Cambridge, UK), CD81 (Abclonal, #A22528, 1:3000, Woburn, MA, USA), TSG101 (Affinity, #DF8427, 1:2000, Cincinnati, OH, USA), P65 (SanYing, #10745-1-AP, 1:3000), CD86 (SanYing, #68674-2-Ig, 1:3000), CD206 (SanYing, #18704-1-AP, 1:3000), iNOS (Affinity, #AF0199, 1:2000).

### 2.2. Validation of Macrophage Targeting in Engineered Exosome Drug Delivery Systems

To validate the successful engineering of exosomes, PKH67 (MCE, 257277-27-3) labeled antiMExo-RIF (10 µg) was co-incubated with macrophages overnight (12), with a control group set for comparison. After fixation with 4% paraformaldehyde, nuclear staining (Servicebio, DAPI, G1012-100ML, Wuhan, China) and cytoskeletal staining (Life iLab, Alexa Fluor 555, AC8L022, Beijing, China) were performed. Subsequently, images were captured using confocal microscopy (Germany, Zeiss LSM 880, Oberkochen, Germany), and quantitative analysis of the average fluorescence intensity and fluorescence area was conducted.

### 2.3. Transcriptome Sequencing and Proteomic Quantitative Analysis

#### 2.3.1. Sample Collection and Processing

After macrophages were altered by the engineered exosome drug delivery system (10 µg, 12), the macrophages were collected. For transcriptomics samples, total RNA was extracted using the TRIzol kit per instructions. Cells were first washed with cold PBS, then lysed with TRIzol reagent. After chloroform treatment and centrifugation, the aqueous phase was separated, and RNA was precipitated with isopropanol and washed with ethanol. The RNA pellet was then dissolved in DEPC-treated water. For proteomics samples, total protein was extracted using cell lysis buffer as instructed. Cells were washed with cold PBS, lysed with protease inhibitor-containing buffer, and sonicated if needed. After centrifugation to remove debris, the supernatant was collected as the total protein extract.

#### 2.3.2. Transcriptome Sequencing and Proteomic Quantitative Analysis

After quality inspection of the RNA samples, transcriptome sequencing was carried out. The RNA libraries were sequenced by OE Biotech, Inc., Shanghai, China. The mass spectrometry analysis of peptides was performed using liquid chromatography–mass spectrometry (Thermo Scientific™, Waltham, MA, USA; Orbitrap™ Waltham, MA, USA; Astral™, Asheville, NC, USA). By comparing the signal intensities of corresponding peptides in different samples, the relative quantification of the proteins corresponding to the peptides was conducted. By comparing the information in the spectral library and integrating it into the corresponding proteins, a quantitative analysis of the relative expression levels of proteins in different samples was achieved.

#### 2.3.3. Bioinformatics Analysis

Gene Ontology (GO) enrichment analysis and Kyoto Encyclopedia of Genes and Genomes (KEGG) pathway analysis were used to analyze the enrichment of the differentially expressed genes in aspects such as biological processes, cellular components, and molecular functions, as well as their enrichment in different signaling pathways, so as to reveal the main biological functions and pathways involved in the changes of gene expression in macrophages after the intervention of the engineered exosome drug delivery system.

For the proteomic data, after log transformation, two parameters were used to jointly evaluate the differences in protein expression between groups, namely, Fold change (FC, calculated from log2FC, where log2(Fold change) = mean of the experimental group—mean of the control group) and the *p*-value obtained through the *t*-test. The differential screening conditions were as follows: *p*-value < 0.05, FC ≥ 2.0 or FC ≤ 1/2.0. Subsequently, GO enrichment analysis and KEGG pathway analysis were used to analyze the enrichment of the differentially expressed proteins.

### 2.4. Verification of Macrophage Polarization and Key Inflammatory Pathways

According to the BCA assay, the exosome concentration was determined to be 1 μg/μL. Subsequently, 10 μg of MExo and antiMExo-RIF were co-cultured with macrophages for 12 h. After incubation, cell samples were collected, and total cellular protein was extracted. The expression levels of the M1 macrophage markers, inducible nitric oxide synthase (iNOS) and CD86, as well as the M2 macrophage marker CD206, were detected by Western blotting technology to evaluate the polarization state of the macrophages. The enzyme-linked immunosorbent assay (ELISA) kits were used to detect the contents of inflammatory factors such as iNOS, IL-1, and tumor necrosis factor-α (TNF-α) in the co-culture supernatant, reflecting the inductive effect of the engineered exosome drug delivery system on the inflammatory response of macrophages. Western blotting was adopted to detect the nuclear translocation of P65, a key protein in the NF-κB pathway, so as to verify whether the engineered exosomes activated the NF-κB pathway.

### 2.5. Macrophage Apoptosis

The Mitochondria Membrane Potential and Apoptosis Detection Kit with Mito-Tracker Red CMXRos and Annexin V-FITC was used to perform apoptosis staining on the macrophages after co-culturing. The live cells with maintained mitochondrial membrane potential were marked by red fluorescence, while the apoptotic cells were marked by green fluorescence.

The experimental data were derived from three independent replicate experiments. Descriptive statistics are presented as mean ± standard deviation. Comparisons between two groups were performed using the independent samples *t*-test, while comparisons among multiple groups were conducted using one-way analysis of variance (ANOVA).

## 3. Results

### 3.1. Extraction, Engineering Modification and Drug Loading of Exosomes

**Figure 1:**
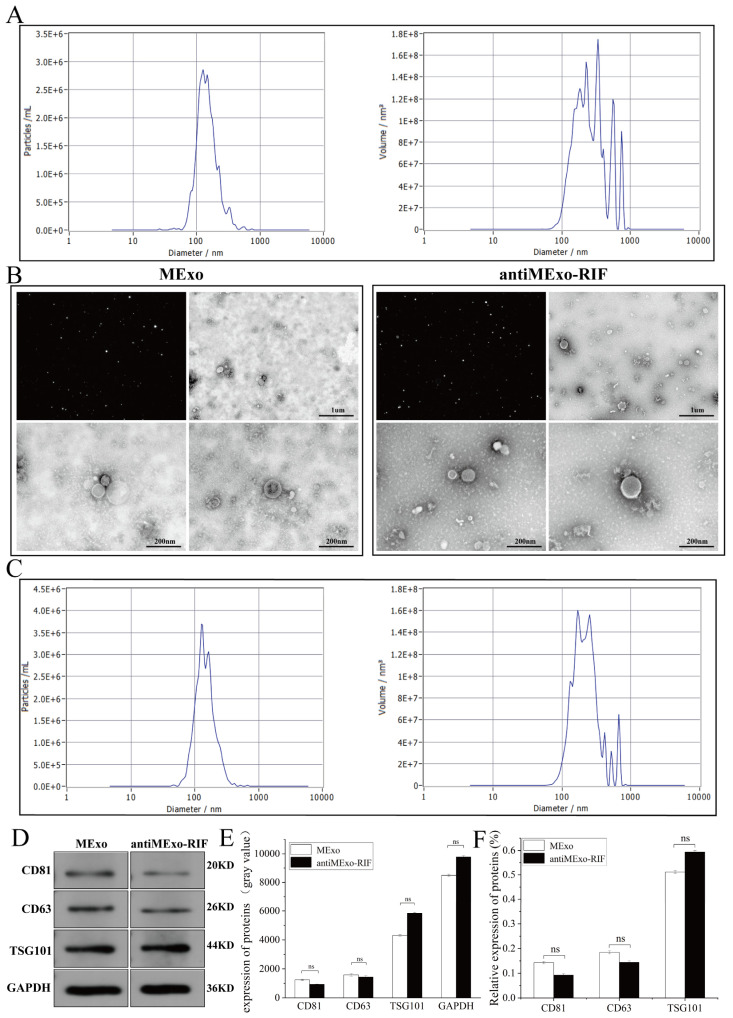
Characterization of macrophage-derived exosomes before and after engineering modification and drug loading. (**A**) Nanoparticle tracking analysis (NTA) of macrophage exosomes (MExo). (**B**) Transmission electron microscopy (TEM) images of macrophage exosomes (MExo) and the engineered exosome drug delivery system (antiMExo-RIF). (**C**) Nanoparticle tracking analysis (NTA) of the engineered exosome drug delivery system (antiMExo-RIF). (**D**–**F**) Western blot results before and after exosome modification. The expression levels (gray value) and relative expression levels of surface characteristic proteins of macrophage exosomes (MExo) and the engineered exosome drug delivery system (antiMExo-RIF). (ns, not significant).

Through transmission electron microscopy (TEM) observation, it could be seen that the exosomes both before and after modification exhibited the typical cup-shaped or round morphology, and their double-membrane structures were clearly distinguishable. The exosome particles in the antiMExo-RIF group were larger than those in the MExo group. In the high-resolution electron microscope images, some regions with low electron density could be observed inside the modified exosomes, which were the rifapentine drugs (Figure 1B).

Nanoparticle tracking analysis (NTA) showed that the particle size distribution of exosomes presented a unimodal normal distribution pattern. The average particle size of the MExo group was 137.8 nm, and that of the antiMExo-RIF group was 139.2 nm, which further confirms that the extracted particles conformed to the particle size characteristics of exosomes. Meanwhile, according to the NTA results, the concentrations of exosomes were approximately 7.2 × 10^7^ particles/mL for the MExo group and 6.3 × 10^7^ particles/mL for the antiMExo-RIF group (Figure 1A,C). The successful loading of rifapentine into the exosomes can be confirmed by their more rounded morphology and the increased particle size. There was no significant change in the relative expression levels of the characteristic proteins of exosomes between the two groups (Figure 1D–F), indicating that the engineering modification and drug loading did not change the structure of exosomes.

### 3.2. Results of the Macrophage Targeting Validation

**Figure 2:**
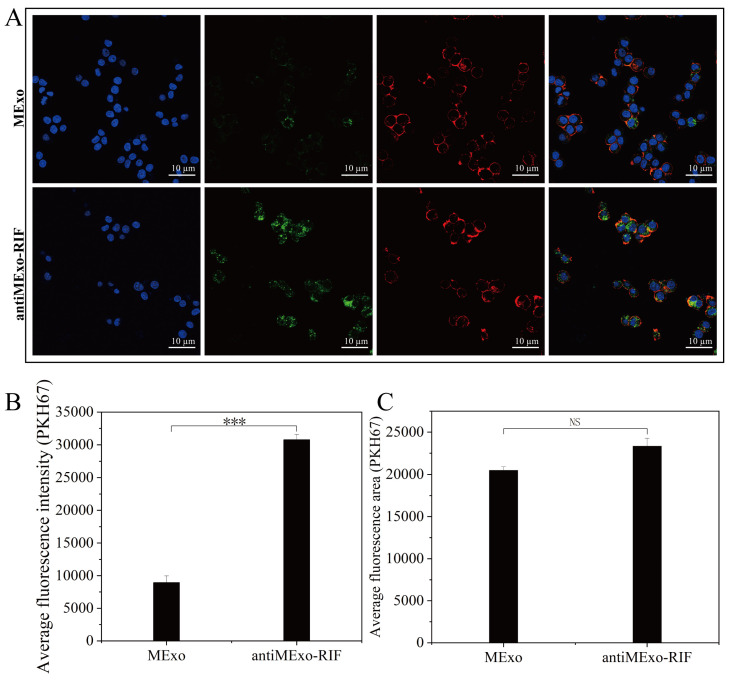
Confocal microscopy images and quantitative fluorescence analysis of macrophages co-incubated with the engineered exosome drug delivery system (antiMExo-RIF). (**A**) Confocal microscopy images showing fluorescence labeling in macrophages after co-incubation with MExo or antiMExo-RIF. Nuclei were stained with DAPI (blue), cytoskeleton with red fluorescence, and exosomes labeled with PKH67 (green). (**B**) Quantitative analysis of PKH67 average fluorescence intensity. (**C**) Quantitative analysis of PKH67 mean fluorescence area. (NS, not significant. ***, *p* < 0.001).

In the MExo group, the green fluorescence signal was weak and scattered, indicating that unmodified exosomes exhibited lower uptake efficiency. In contrast, the antiMExo-RIF group showed significantly enhanced green fluorescence signals concentrated intracellularly, overlapping with nuclear and cytoskeletal structures, demonstrating that the modified exosomes were more effectively targeted and internalized by macrophages, thereby validating the success of the engineered targeting capability (Figure 2A). The average fluorescence intensity of the antiMExo-RIF group was significantly higher than that of the MExo group (*p* < 0.05), further confirming a marked increase in antiMExo-RIF uptake by macrophages (Figure 2B). No significant difference was observed in the mean fluorescence area (PKH67) between the two groups (*p* > 0.05), suggesting that although antiMExo-RIF uptake increased, its intracellular distribution remained localized to specific regions, consistent with the confocal imaging observations (Figure 2C).

### 3.3. Results of Transcriptome Sequencing

#### 3.3.1. Differential Gene Expression Analysis

**Figure 3:**
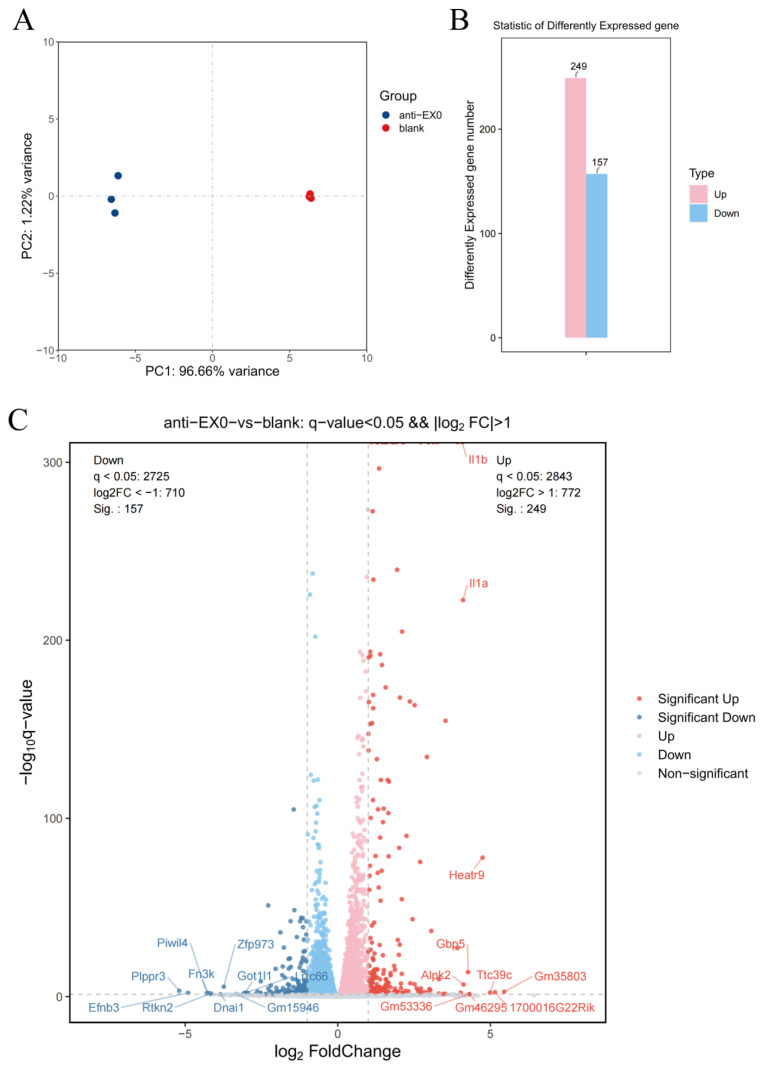
Transcriptomics of macrophages after intervention with the exosome drug delivery system (antiMExo-RIF). (**A**) Principal component analysis (PCA). (**B**) Comparison of the expression levels of up-regulated and down-regulated differential genes. (**C**) Volcano plot of differential gene expression. The up-regulated genes are distributed in the upper right region of the plot, and the down-regulated genes are in the lower left region. Each dot represents a gene, the size of the dot indicates the level of gene expression, and the color represents the significance level of differential gene expression.

Principal component analysis was performed on the samples, and it was found that there was a large difference between the two groups. PC1 explained 96.66% of the variance, while PC2 explained 1.22% of the variance (Figure 3A). Transcriptome sequencing analysis revealed that, compared with the control group, the expressions of 406 genes in the intervention group changed significantly. Among them, there were 249 up-regulated genes and 157 down-regulated genes (Figure 3B). Volcano plots have been drawn for these differentially expressed genes, allowing for a visual overview of the overall situation of gene expression changes (Figure 3C). Among them, IL-1α and IL-1β had the highest significance of gene expression differences, indicating that the treatment with engineered exosomes had a significant impact on the expression of macrophage immune-related genes.

#### 3.3.2. Gene Enrichment Analysis

**Figure 4:**
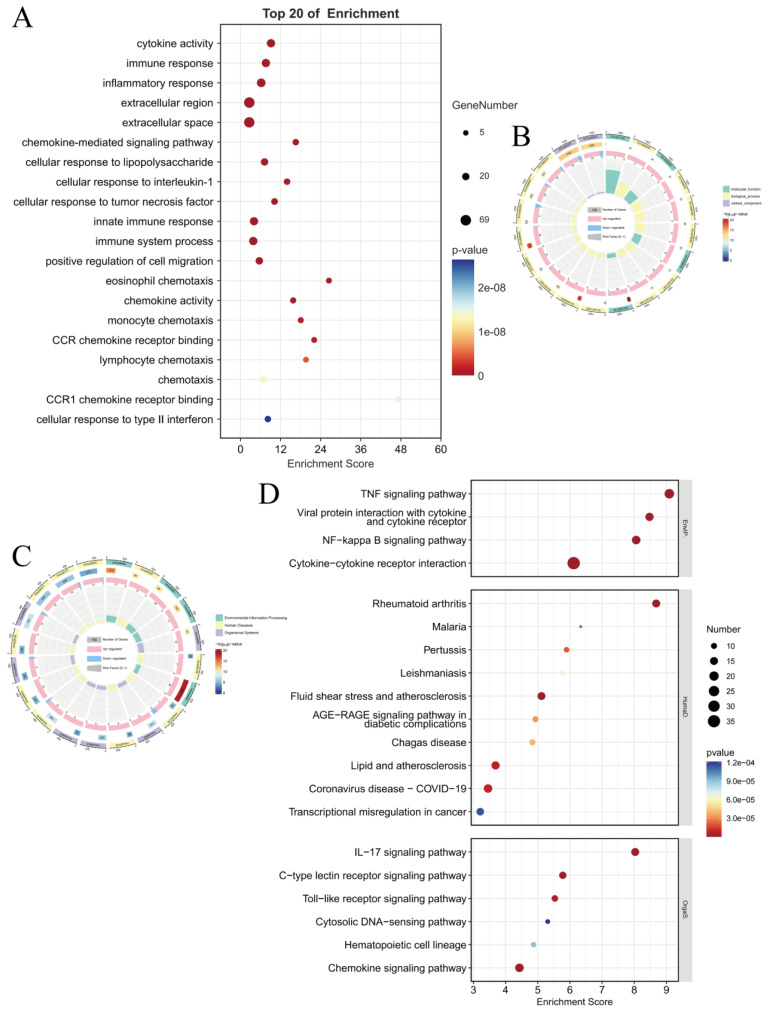
(**A**) Bubble plot and (**B**) circle diagram of the top 20 Gene Ontology (GO) enrichments in the transcriptomics of macrophages after intervention with the exosome drug delivery system (antiMExo-RIF). (**D**) Bubble plot and (**C**) circle diagram of the top 20 Kyoto Encyclopedia of Genes and Genomes (KEGG) pathway enrichments.

Through Gene Ontology (GO) enrichment analysis, it was found that these differentially expressed genes were significantly enriched in three aspects: biological processes, cellular components, and molecular functions (Figure 4A,B). In terms of biological processes, they were mainly enriched in “inflammatory response”, “cytokine activity”, “immune response”, “innate immune response”, and “immune system process”. In terms of cellular components, the differentially expressed genes were enriched in “extracellular region” and “extracellular space”. In terms of molecular functions, they were mainly enriched in functions such as “eosinophil chemotaxis”, “chemokine activity”, “monocyte chemotaxis”, and “CCR chemokine receptor binding”. These functions are closely related to macrophage recognition, the uptake of exosomes, and subsequent signal transduction and functional regulation.

KEGG pathway analysis showed (Figure 4C,D) that the differentially expressed genes were mainly enriched in “TNF signaling pathway”, “Viral protein interaction with cytokine and cytokine receptor”, “NF-kappa B signaling pathway”, and “Cytokine-cytokine receptor interaction”. Among them, the largest number of differentially expressed genes was found in “Cytokine–cytokine receptor interaction”, indicating that a large number of differentially expressed genes were involved in this process after macrophages phagocytosed the engineered exosome drug delivery system. And these pathways with the most obvious enrichment were closely related to apoptosis. In the KEGG pathway enrichment track, the one with the highest enrichment degree and significant up-regulation was mmu04060 (Cytokine–cytokine receptor interaction, PopHits = 293) in “Environmental Information Processing”. Many up-regulated genes in this pathway (Ccl2, IL-1α, IL-1β, TNF-α) all reflected the effective activation of the macrophage inflammatory pathway by the engineered exosome drug delivery system.

### 3.4. Results of Proteomic Quantitative Analysis

#### 3.4.1. Protein Identification and Quantification Results

**Figure 5:**
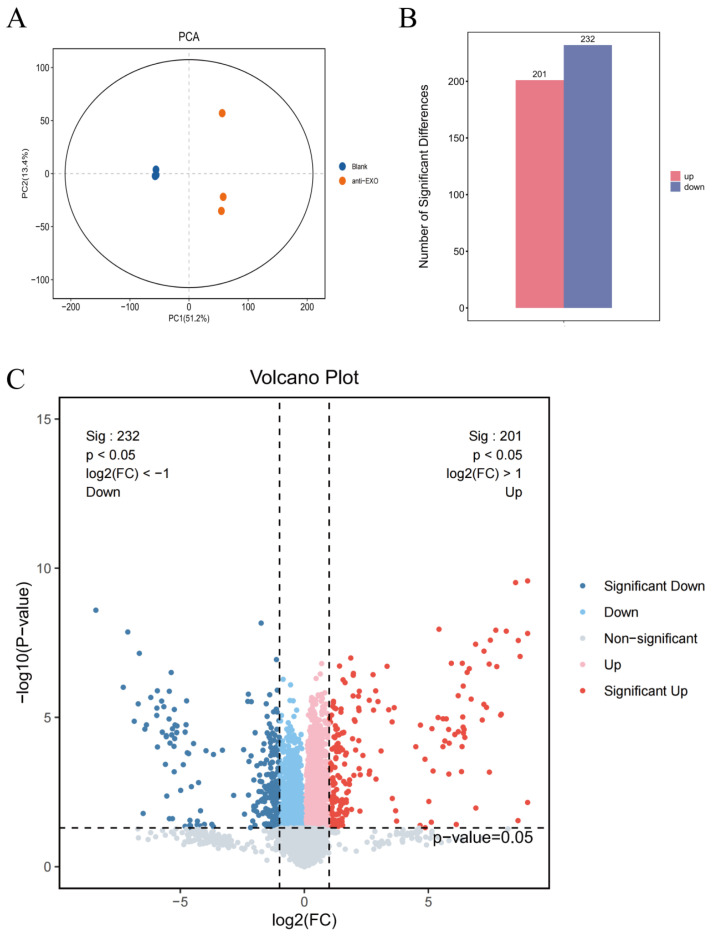
Proteomics of macrophages after intervention with the exosome drug delivery system (antiMExo-RIF). (**A**) Principal component analysis (PCA). (**B**) Comparison of the expression levels of up-regulated and down-regulated differential genes. (**C**) Volcano plot of differential gene expression.

Through data independent acquisition (DIA), proteomic quantitative analysis identified a total of 7478 proteins and 108,046 peptides. Principal component analysis revealed a large difference between the two groups, with PC1 explaining 51.2% of the variance and PC2 explaining 13.4% of the variance (Figure 5A). Among them, 433 proteins had significant differences between the intervention group and the control group, with 201 proteins being significantly up-regulated and 232 proteins being significantly down-regulated (Figure 5B). Volcano plots were drawn for these differentially expressed proteins to visually display the overall situation of protein expression changes (Figure 5C).

#### 3.4.2. Differential Protein Function Analysis

**Figure 6:**
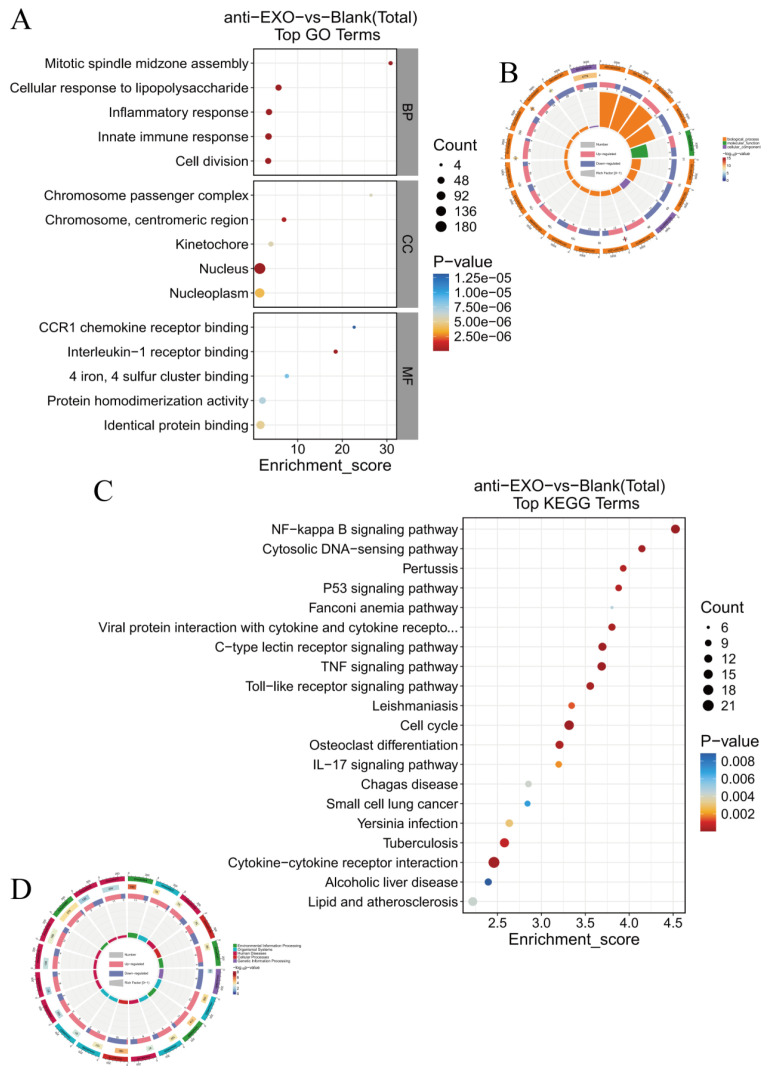
(**A**) Bubble plot and (**B**) circle diagram of the top 20 GO enrichments in the proteomics of macrophages after intervention with the exosome drug delivery system (antiMExo-RIF). (**C**) Bubble plot and (**D**) circle diagram of the top 20 KEGG pathway enrichments.

Through Gene Ontology (GO) enrichment analysis, it was found that these differential proteins were significantly enriched in three aspects: biological processes, cellular components, and molecular functions (Figure 6A,B). In terms of biological processes, the expression levels of some proteins related to “Inflammatory response” (such as P20491:Fcer1g, Q62178:Sema4a, Q9R0Q8:Clec4e, Q9Z2H6:Clec4d) had changed, which might have affected the initial interaction between macrophages and exosomes and promote or inhibited the adhesion of exosomes to macrophages. In terms of cellular components, the expression of proteins related to “Nucleus” (such as P01582:Il1a, P10749:Il1b, P25085:Il1rn, Q62406:Irak1, Q8R4K2:Irak4, Q9EQU3:Tlr9, Q9JLA2:Il36a) had changed, indicating that engineered exosomes might affect the immune function of macrophages by regulating the expression of these proteins. In terms of molecular functions, the changes in the expression levels of proteins related to “Interleukin-1 receptor binding” (such as E9PVX6:Mki67, O70126:Aurkb, O70201:Birc5, Q07832:Plk1) further confirmed the regulatory effects of engineered exosomes on the immune function of macrophages.

KEGG enrichment analysis showed that the most significant enrichment and the highest scores were in “Cytokine–cytokine receptor interaction” and “NF-kappa B signaling pathway”, followed by “TNF signaling pathway” (Figure 6C,D). In the KEGG pathway enrichment track, the one with the highest enrichment degree and most significant up-regulation was mmu04060 (Cytokine–cytokine receptor interaction, PopHits = 276). These results are consistent with and mutually verified by the transcriptome analysis results, further revealing the influence mechanism of the engineered exosome drug delivery system on the immune effect of macrophages at the protein level.

**Figure 7:**
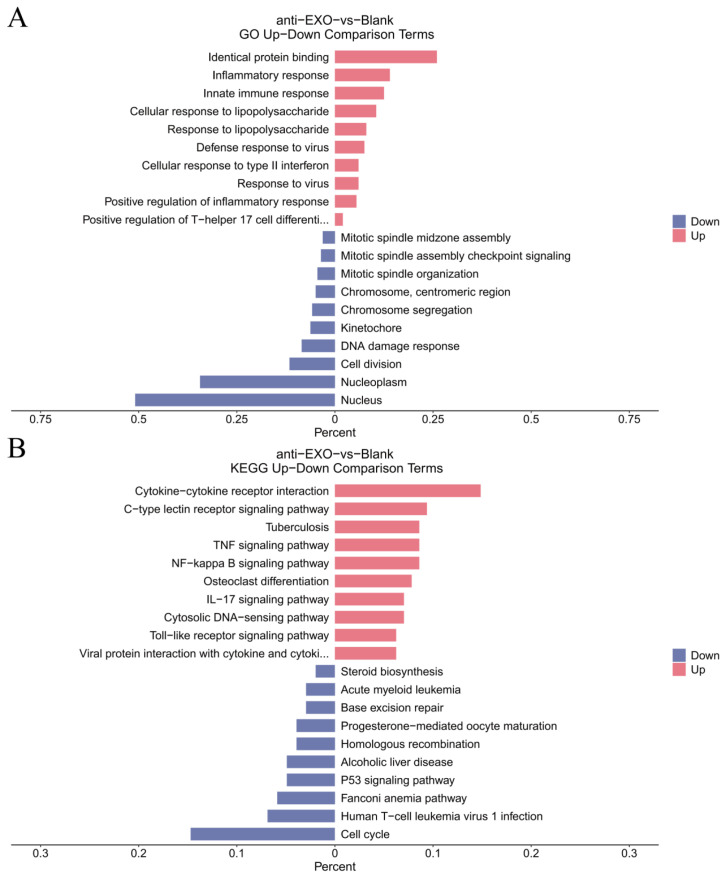
(**A**) Comparison of protein functions in GO enrichment and (**B**) comparison of pathways in KEGG enrichment for up-regulation and down-regulation in the proteomics of macrophages after intervention with the exosome drug delivery system (antiMExo-RIF).

We conducted GO and KEGG enrichment analyses on the most significantly up-regulated and down-regulated proteins in the differential protein expression and made a comparison (Figure 7A,B). It was found that the number of differentially expressed proteins ranked third in “Tuberculosis”, and our research object was also “Tuberculosis”, which indirectly verifies our research hypothesis and provides a theoretical basis for the subsequent construction of a bone tuberculosis model.

### 3.5. Combined Analysis of Transcriptomics and Proteomics

**Figure 8:**
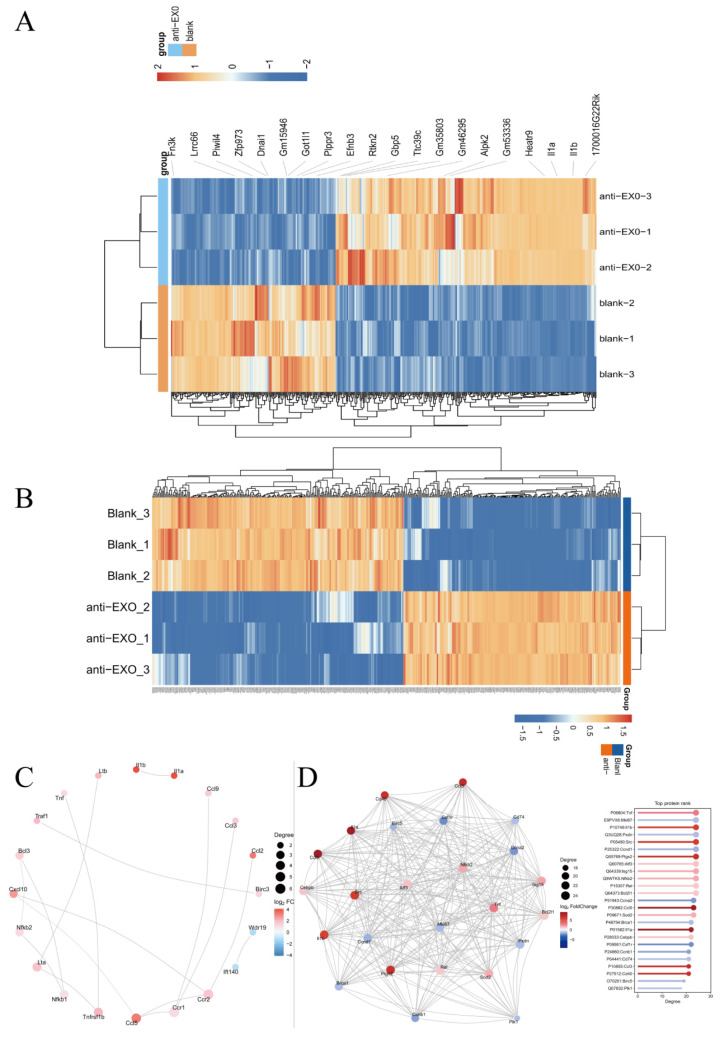
(**A**) Heat map of differential gene expression in transcriptomics, (**B**) heat map of differentially expressed proteins in proteomics, (**C**) network co-occurrence diagram of differential genes, and (**D**) network co-occurrence diagram of differential proteins of macrophages after intervention with the exosome drug delivery system (antiMExo-RIF).

Abundance analyses were conducted on these differential genes and proteins, and heat maps were drawn to visually display the changes in the expression levels of the differential proteins in different samples (Figure 8A,B). In the heat maps, each row represents a differential protein, and each column represents a sample. The depth of the color indicates the level of gene or protein expression. Through the heat maps, the differences in the gene or protein expression patterns between the intervention group and the control group, as well as the changing trends in the expression levels among different differential genes or proteins, can be clearly seen. Meanwhile, visual analyses of network co-occurrence were carried out respectively for the genes and proteins with the highest differential expression (Figure 8C,D). It could be intuitively seen from the figures that the genes and proteins of IL-1α and IL-1β had the highest enrichment scores, indicating that IL-1α and IL-1β played a key role in the immune effect after the engineered exosome drug delivery system entered macrophages.

**Figure 9:**
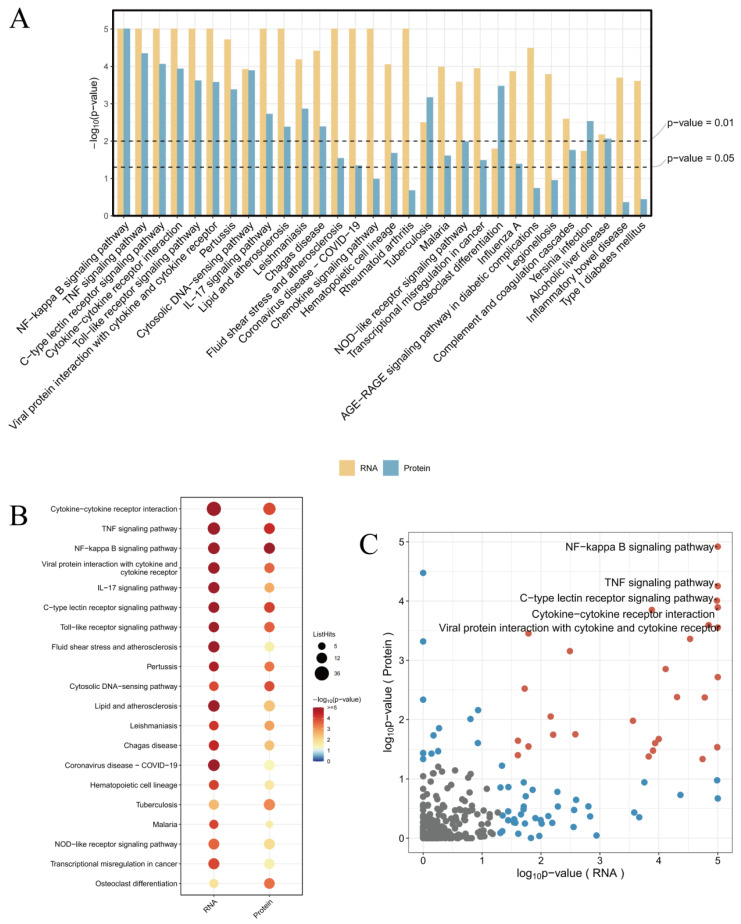
Combined analysis of transcriptomics and proteomics of macrophages after intervention with the exosome drug delivery system (antiMExo-RIF). (**A**) Bar chart, (**B**) bubble chart, (**C**) volcano plot.

Subsequently, KEGG combined enrichment analyses were performed on the key pathways regulated by the differential genes and proteins, and bar charts, bubble charts, and volcano plots were drawn (Figure 9A–C). It could be intuitively seen from these figures that the NF-kappa B signaling pathway, TNF signaling pathway, and Cytokine–cytokine receptor interaction had the highest enrichment scores in the differential gene and protein expressions, indicating that the engineered exosome drug delivery system mainly regulated the immune effect of macrophages through these three pathways. The significantly differentially expressed genes, proteins (IL-1α, IL-1β), and signaling pathways (NF-kappa B signaling pathway, TNF signaling pathway, Cytokine–cytokine receptor interaction) screened out by transcriptomics and proteomics provided a theoretical basis for the subsequent verification steps.

### 3.6. Verification of Macrophage Polarization and Key Inflammatory Pathways

**Figure 10:**
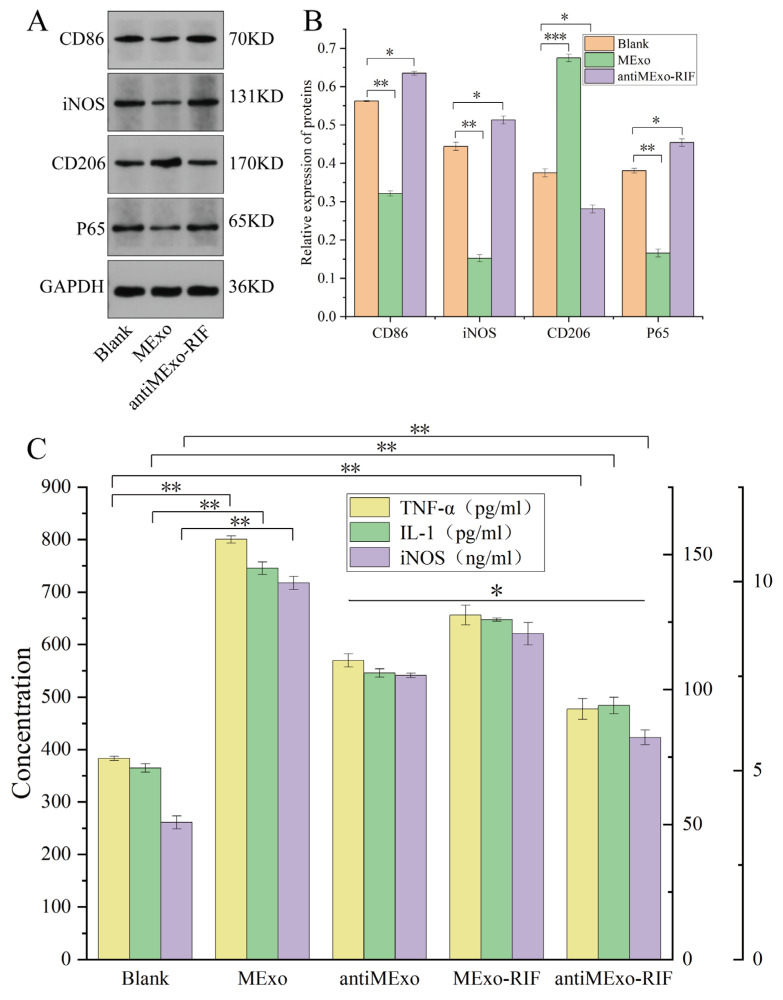
(**A**,**B**) Western blotting (WB) detection of polarization markers and P65 protein in the NF-κB signaling pathway, (**C**) quantitative detection (ELISA) of inflammatory factors TNF-α, IL-6, and iNOS of macrophages after intervention with the exosome drug delivery system (antiMExo-RIF). (*, *p* < 0.05. **, *p* < 0.01. ***, *p* < 0.001).

Western blotting (WB) was used to study the polarization of macrophages after the intervention of the engineered exosome drug delivery system. The results show that compared with the blank group, the gray value of the CD86 protein band in the antiMExo-RIF group was significantly increased (*p* < 0.05), indicating that its expression level was significantly up-regulated. This suggests that during the polarization process towards classically activated (M1) macrophages, CD86, as a co-stimulatory molecule, was highly expressed, which was beneficial for antigen presentation and the exertion of immune activation functions. As for CD206, a marker of M2 macrophages, the experimental results clearly show that the gray value of its protein band in the antiMExo-RIF group was significantly lower than that in the blank group (*p* < 0.05). The above results indicate that the engineered exosome drug delivery system (antiMExo-RIF) can induce macrophages to polarize towards M1 (Figure 10A,B).

The WB results of inducible nitric oxide synthase (iNOS) show that after macrophages in the antiMExo-RIF group polarized towards M1, the expression level of iNOS protein increased sharply, and the difference compared with the blank group was statistically significant (*p* < 0.05). Moreover, its ELISA results are consistent with those of WB. Corresponding to its functional characteristics of generating a large amount of nitric oxide to participate in sterilization and inflammatory responses, it is a key enzyme for M1 macrophages to exert pro-inflammatory effects (Figure 10A,B).

To verify the activation of the NF-kappa B signaling pathway, TNF signaling pathway, and Cytokine–cytokine receptor interaction, the key factors in these pathways were detected. When detecting the P65 subunit of the nuclear transcription factor NF-κB family, it was found that the total protein expression level of P65 in the antiMExo-RIF group had an upward trend (*p* < 0.05), suggesting that the NF-κB signaling pathway was efficiently activated during the M1 polarization process. After phosphorylation, P65 translocated into the nucleus and drove the transcription and expression of a series of downstream pro-inflammatory genes. The ELISA results show that the secretion amount of TNF-α in the antiMExo-RIF group was far higher than that in the blank group, and the difference was statistically significant (*p* < 0.05). As a classic pro-inflammatory cytokine, its high secretion amount accurately reflects the core role of M1 macrophages in releasing a large amount of TNF-α during the inflammation initiation and immune activation stages to recruit immune cells and amplify the inflammatory response. The concentration of interleukin-1 (IL-1) in the antiMExo-RIF group was significantly higher than that in the blank group (*p* < 0.05), which is in line with the functional mode of M1 macrophages in the activated state, where they release IL-1 in coordination with cytokines such as TNF-α to widely stimulate the proliferation and differentiation of immune cells and the secretion of inflammatory mediators, expanding the “battlefield” of inflammation (Figure 10C).

### 3.7. Macrophage Apoptosis

**Figure 11:**
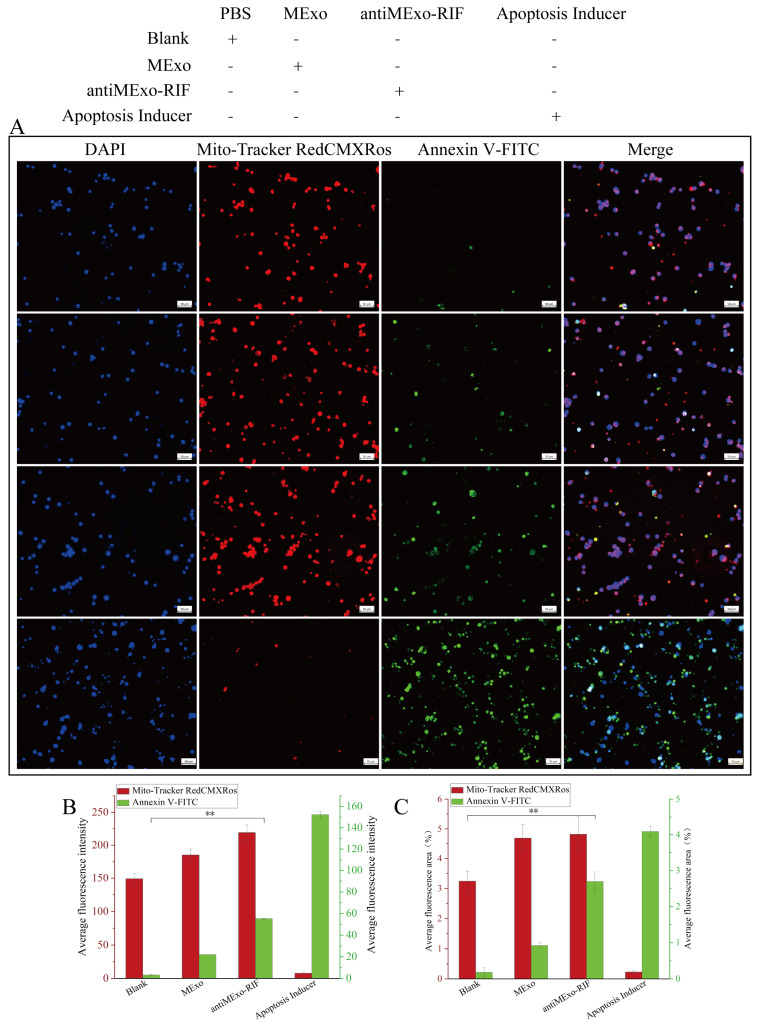
(**A**) Detection of cell apoptosis using the Mitochondria Membrane Potential and Apoptosis Detection Kit with Mito-Tracker Red CMXRos and Annexin V-FITC after macrophages had been supplemented with the exosome drug delivery system (antiMExo-RIF). (**B**,**C**) Quantification of the average fluorescence intensity and fluorescence area. Scale bar: 50 µm. (**, *p* < 0.01).

After the intervention of the engineered exosome drug delivery system (antiMExo-RIF), the situation of cell apoptosis changed significantly. To improve the logic of the experimental design, an apoptosis induction group was added as a positive control (Figure 11A). The results show that the proportion of Annexin V-FITC-positive cells in the antiMExo-RIF group increased significantly, and both the average fluorescence intensity and the fluorescence area were significantly higher than those in the blank group (*p* < 0.05) (Figure 11B,C). This indicates that after the intervention of the engineered exosome drug delivery system, the proportion of apoptotic cells increased significantly.

## 4. Discussion

In this study, a macrophage-derived engineered exosome drug delivery system was successfully constructed to target macrophages, induce macrophage polarization towards M1, exert its pro-inflammatory and antibacterial effects, activate relevant inflammatory pathways to further promote the expression of inflammatory factors, and ultimately induce macrophage apoptosis, thereby destroying the living environment of Mycobacterium tuberculosis and achieving an antibacterial effect. From a molecular perspective, the mechanisms behind this phenomenon involve multiple aspects.

From the perspective of immune recognition, CD47, as an important “don’t eat me” signal molecule, interacts with SIRPα on the surface of macrophages and inhibits the phagocytic activity of macrophages under normal physiological conditions [13,14]. When we engineered the RAW264.7 exosomes with anti-CD47 antibody, the antibody bound to CD47 or related antigens on the surface of exosomes, effectively blocking this inhibitory signal. This is like removing a barrier that prevents macrophages from “eating”, enabling macrophages to recognize and phagocytose exosomes more actively [15,16]. The core principle of this engineered exosome-based drug delivery system resembles the Trojan Horse strategy from ancient Greek mythology. It involves encapsulating therapeutic agents (the soldiers) within exosomes (the wooden horse). Through engineered surface modifications, these exosomes can mislead macrophages (the city of Troy) into keeping their cell membrane channels open, thereby enabling entry into the cellular interior and achieving sustained drug release effects.

The highly differentially expressed genes and proteins screened by transcriptomics and proteomics were consistent and mutually verified. In particular, IL-1α and IL-1β, as important pro-inflammatory cytokines, play a central role in the immune activation of macrophages. They can induce the activation of multiple immune cells and promote the release of inflammatory mediators, such as tumor necrosis factor-α (TNF-α) and interleukin-6 (IL-1) [17], which was also proven by the ELISA results. After the engineered exosome drug delivery system entered macrophages, the differential expressions of IL-1α and IL-1β indicated that they initiated a series of subsequent inflammatory cascade reactions in macrophages, including M1 polarization of macrophages and the activation of inflammatory pathways. Since IL-1α and IL-1β can regulate the phagocytic ability, antigen presentation function, and cytokine secretion pattern of macrophages [18], they can enhance the phagocytosis of Mycobacterium tuberculosis by macrophages, promote antigen processing and presentation, and thus activate the adaptive immune response.

The combined analysis of transcriptomics and proteomics can comprehensively reveal the panorama of gene expression. By leveraging the complementary nature of transcriptomics and proteomics, more complete gene expression information can be obtained, enhancing the accuracy and reliability of the research. The mutual validation of multi-omics data helps reduce false-positive results. The study found that the enriched pathways showed consistency between transcriptomics and proteomics, particularly in the NF-κB signaling pathway, TNF signaling pathway, and Cytokine–cytokine receptor interaction, which were the most significant pathways (Figure 9A). This indicates that these pathways are regulated by antiMExo-RIF at both the gene and protein expression levels. The bubble chart (Figure 9B) shows that these pathways exhibit higher significance at the RNA level and involve a larger number of genes, while at the protein level, the significance is lower and fewer proteins are involved. This discrepancy may reflect the complex relationship between transcriptional regulation and translational regulation, as well as potential additional regulatory mechanisms at the protein level. However, the ranking of the number of genes and proteins involved in these pathways remains consistent. From the volcano plot (Figure 9C), it can be observed that most signaling pathways show some correlation in significance between the RNA and protein levels, but some pathways deviate significantly from the diagonal. For example, the NF-κB signaling pathway, TNF signaling pathway, and Cytokine–cytokine receptor interaction exhibit high significance at both RNA and protein levels, and are located in the upper right quadrant of the scatter plot, indicating that they are tightly regulated at both levels.

The NF-κB signaling pathway is one of the key pathways that regulate immune and inflammatory responses [19]. It can be activated by a variety of stimuli in macrophages, including pathogen-associated molecular patterns (PAMPs), damage-associated molecular patterns (DAMPs), and cytokines, etc. [20]. In this study, the activation of the NF-κB signaling pathway has been verified. This may be caused by the differential expression of cytokines such as IL-1α and IL-1β after the engineered exosome drug delivery system enters macrophages. The activation of the NF-κB signaling pathway can lead to the expression of a series of downstream genes, further amplifying the inflammatory response [21]. The engineered exosome drug delivery system may regulate the functions of macrophages by affecting the upstream regulatory factors or downstream effector molecules of the NF-κB signaling pathway. For example, specific components in exosomes may bind to receptors on the surface of macrophages to activate or inhibit the NF-κB signaling pathway. The specific mechanism still requires further in-depth research. Studying the mechanism of the NF-κB signaling pathway in the response of macrophages to the exosome drug delivery system can provide a basis for designing more effective treatment strategies. By regulating the activity of the NF-κB signaling pathway, the degree of the inflammatory response can be controlled and the therapeutic effect of the exosome drug delivery system can be improved.

The TNF signaling pathway is also an important immune and inflammatory regulatory pathway. TNF-α is a key molecule in this pathway, which can be produced by immune cells such as macrophages and plays an important role in the initiation and maintenance of the inflammatory response [22]. The ELISA results also show that its expression level was significantly increased. The activation of the TNF signaling pathway may be correlated with the differential expression of IL-1α and IL-1β as well as the activation of the NF-κB signaling pathway [23]. It can further activate other immune cells, amplify the inflammatory response, and also regulate the functions of macrophages, including multiple biological effects such as macrophage activation, cytokine secretion, and cell death [24,25]. Subsequent experiments verified that macrophages polarized towards M1 and induced the secretion of multiple cytokines (such as IL-1, iNOS) to participate in the pro-inflammatory process, ultimately inducing macrophage apoptosis.

Cytokine–cytokine receptor interaction exerts its biological functions by binding cytokines to receptors on the cell surface, and is one of the key aspects of immune regulation. It can regulate the activation, proliferation, differentiation, and functions of immune cells [26,27]. In this study, the increased secretion of differentially expressed cytokines (such as IL-1α, IL-1β, IL-6, iNOS, and TNF-α, etc.) led to an increase in specific binding with their receptors, which could trigger a cascade amplification of signal transduction. For example, after IL-1α and IL-1β bind to IL-1R1/IL-1RAcP, they activate the nuclear factor κB (NF-κB) and mitogen-activated protein kinase (MAPK) signaling pathways, and then induce the production of more cytokines. This cascade amplification effect can rapidly amplify the immune response, but it may also lead to excessive inflammatory responses [28,29,30]. In-depth research into the role of Cytokine–cytokine receptor interaction in the response of macrophages to the exosome drug delivery system can provide clues for designing more effective treatment strategies [31]. Combinations of multiple cytokines and receptors form a complex network. Different cytokines can bind to receptors simultaneously or sequentially, influencing each other’s signal transduction. This complex interaction determines the functional state of macrophages and the direction of the immune response.

The interaction between cytokines (TNF itself is also a cytokine) and their receptors can initiate or regulate the TNF signaling pathway [32]. Meanwhile, the downstream signals activated by cytokine–receptor interactions can converge into the NF-κB signaling pathway. By regulating the activity of NF-κB, they participate in multiple physiological and pathological processes such as immune responses and inflammatory responses. These three pathways are intertwined and jointly play extremely important roles in numerous crucial cellular activities, such as cell signal transduction, immune responses, and inflammatory responses [33].

Although this study has achieved a series of meaningful results, there are still some limitations. Although transcriptome sequencing and proteomic quantitative analyses have revealed changes in the expression of a large number of genes and proteins, the functional verification of some key molecules is not yet in-depth enough. Although we have found that many genes and proteins have undergone significant changes during the process of engineered exosomes interfering with macrophages, their specific action mechanisms in the entire immune effect process, such as whether there are direct interactions and whether there are upstream and downstream regulatory relationships, still need to be further studied in detail through molecular biology experiments (such as gene knockout, overexpression experiments, and protein–protein interaction experiments). Only by understanding the functions of these key molecules more deeply can we comprehensively elaborate the molecular mechanism of the interaction between engineered exosomes and macrophages and provide a more solid theoretical basis for their application in drug delivery systems.

## 5. Conclusions

In this study, an engineered exosome drug delivery system was constructed. Through transcriptome sequencing and proteomic quantitative analysis, the related molecular mechanisms were initially revealed and relevant verifications were carried out. After the engineered exosome drug delivery system enters macrophages, it can stimulate M1 polarization, secrete multiple inflammatory factors to participate in the pro-inflammatory process, and activate multiple signaling pathways to generate cascade reactions and amplify the inflammatory response, thereby inducing macrophage apoptosis. These results provide a theoretical basis and new ideas for the application of exosomes in fields such as immunotherapy, and also offer valuable data for further in-depth research on the interaction between exosomes and macrophages. Future research will focus on the functional verification of key molecules and the study of in vivo models to better expand the application prospects of this research result.

## Data Availability

The raw sequence data reported in this paper have been deposited in the Genome Sequence Archive, China National Center for Bioinformation/Beijing Institute of Genomics, Chinese Academy of Sciences (GSA: CRA021372, OMIX: OMIX008307) that are publicly accessible at https://ngdc.cncb.ac.cn/gsa.
